# Spiritual practices as coping with mothers of children with attention-deficit hyperactivity disorder: a qualitative explorative study

**DOI:** 10.1186/s40359-024-02331-2

**Published:** 2025-01-02

**Authors:** K. V. G. S. G. Vithana, T. A. Asurakkody

**Affiliations:** 1 National Hospital, Galle, Sri Lanka; 2 Department of Fundamental Nursing, Faculty of Nursing, University of Colombo, Colombo, Sri Lanka

**Keywords:** Spiritual, Practices, Religious, Coping, Mothers, ADHD

## Abstract

**Background:**

Mothers of children with ADHD struggle with everyday challenges that exceed from the signs and symptoms of ADHD. They have applied different coping mechanisms, comprising spiritual practice commitments. The study aims to explore spiritual practices as a coping mechanism for mothers of children with ADHD.

**Method:**

Qualitative exploratory research methodology was conducted with 22 mothers who were selected via purposive sampling. Semi-structured interviews were conducted, and the data were analysed via thematic analysis.

**Results:**

The findings were discussed with three main themes and six subthemes:01) cognitive appraisal—thinking with faith and belief about higher power; 2) behavioral practices—individual practices and group approaches; and 3) support from religious leaders—common support with religious concepts and specific personnel support.

**Conclusions:**

This study discussed the religious commitment as coping of mothers, who used them as coping mechanisms while caring for a child with ADHD. They found a variety of culturally bound religious practice methods that were reported to be perceived as effective for their mental well-being.

## Introduction

Attention deficit hyperactivity disorder (ADHD) is a neurobehavioral illness that affects children and adults [1, 2]. ADHD symptoms include inattention (not having the ability to keep view), hyperactive behavior (extravagant motion that is not suitable for the setting) and impulsive actions (expeditious activities that can occur in moments without a thought process). ADHD is a habitual and debilitating mental disorder that influences personnel in different aspects of life, including educational and professional accomplishments, interpersonal connections, and daily living activities [2]. ADHD influences a child’s mental well-being and development from a cognitive perspective [3]. It is estimated that 8.4% of children and 2.5% of adults have ADHD [4, 5]. The prevalence of ADHD in Sri Lanka is reported to be 6.5%, with the condition commonly observed in seven- to eight-year-old children [6].

Mothers of children with ADHD struggle with challenges related to excessive symptoms of ADHD. Struggling and experiencing are very important aspects for these mothers [7]. In the Sri Lankan context, most mothers and fathers who have a child with ADHD face challenges and fulfil the requirements of their child [6]. Caring for a family member with ADHD is a noticeable challenge during daily activities [7]. Caring for a family member can give rise to various issues affecting both the family and the well-being of the caregiver [8]. Few facilities are reported for child and adolescent mental health care settings and aspects of community mental health nursing in Sri Lanka [9].

Mothers practice different methods to cope with stress and anxiety [10]. Some parents with disabled children have low adaptive coping techniques in the long term [11]. Spiritual benefits include situational coping methods such as empowering hope, sociological support, and coping with pain [12]. Spiritual practices emerge during life crisis events such as illness, stress, and fear of death, prompting individuals to question the meaning of life. Spirituality often increases in mothers of children with developmental disabilities who feel hopeless [13]. Spirituality involves personal moral values and religious beliefs that can be used to support mothers in understanding and accepting their current situation. Interventions aimed at religiously reducing stress among mothers who have intellectually disabled children are necessary and virulent. Mothers who are engaging in religious practices and rely on God to decrease their mental stress and increase their level of mental health [14].

### Theoretical aspect of the study

The current study is most related to the meaning-making model, which is a psychological theory that focuses on the distress experienced by individuals when there is a discrepancy between the meaning they give to a particular situation (appraisal) and their overarching system of beliefs, goals, and subjective sense of meaning formed from early experiences system (global). When considering the above model, individuals try to decrease their inconsistency and distress through retrospective meaning-making [15].

The meaning-making model includes spiritual and religious meaning-making to reduce distress. Spirituality has the ability to address all features of global meaning, informing beliefs such as the nature of God, karma, humanity, control, and destiny, and offering inspiration and primary goals and guidelines for achieving those goals, as well as a heartfelt understanding of purpose and mattering [16].Within this framework, important to explore mothers’ spiritual practices as a coping for living with their ADHD child.

### Objectives

Current research focused on exploring spiritual practices as a coping mechanism for mothers of children with ADHD in child and adolescent clinics at Teaching Hospital Galle, Sri Lanka.

## Methodology

### Study design

The study design is a qualitative exploratory design. The qualitative approach assists in developing and enhancing the knowledge of the deep experiences of individuals [17].

### Study setting

The study was conducted at the child and adolescent clinic at Teaching Hospital Galle, Sri Lanka.

### Sampling

Study participants were recruited from the child and adolescent clinic at Teaching Hospital Karapitiya in Galle, Sri Lanka. Twenty-two mothers were selected via purposive sampling. The inclusion criteria were having a child with ADHD and specific attributes of the mothers. The child had to be under 12 years of age and either make their first visit to the clinic or currently receive treatment for symptoms. The mothers' willingness to explore their practices was considered. Health care workers and individuals with a history of mental illness were excluded. The recruitment of mothers continued until data saturation was reached [18].

### Data collection

**Fig. 1:**
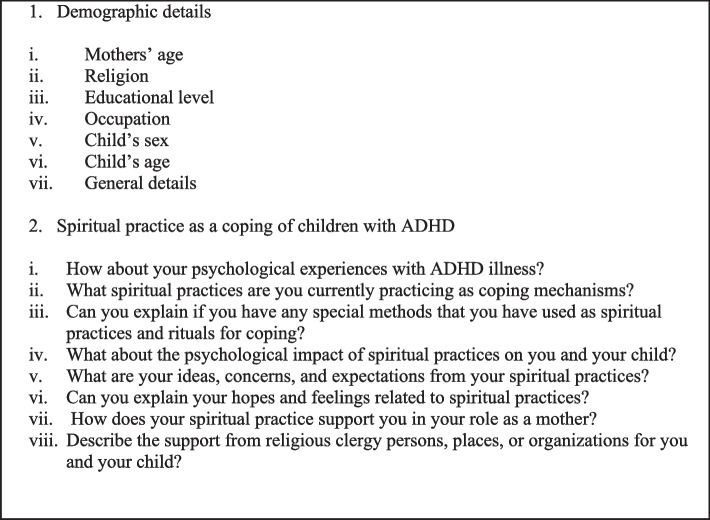
Interview guide

Data collection was performed through face-to-face interviews in a private place in the clinic from April to May 2023 via a semi-structured interview guide. The semi-structured interview guide did not have direct asking questions from the participants; however, it was helpful to keep in the topics that required to be contemplated during interviews (Fig. 1). The study interview guide was developed by the principal investigator on the basis of a comprehensive review of the past literature and was accomplished through conversation among both researchers. Interviews with mothers were managed in a narrative method and reflectively. Interview questions were explorative and used open-ended words such as 'How about you feel?', 'What are your thoughts?', and 'Can I get your input on this?' for the purpose of provoking study mothers to explain their spiritual practices in depth. The principal investigator who conducted the interview questions was careful to minimize any potential impact of his experience as a registered mental health nurse. A total of 17 participants were individually interviewed until the point of data saturation. Twenty-two mothers were individually interviewed face-to-face in both the Sinhala and English languages until data saturation was reached. The interviews were approximately 30 to 45 min in length. Prior permission was obtained to record the interviews, which were then recorded on audio tapes. Next, the interviews were transcribed verbatim and translated from Sinhala to English by the principal investigator. Finally, both researchers reviewed the transcripts, and any inconsistencies in translation were addressed through discussion.

### Data analysis

The data analysis was completed via thematic analysis. Thematic analysis is widely used and is considered one of the validated methods for qualitative research [19]. It involves seven steps, including becoming familiar with the data, coding, finding and reviewing themes, defining themes, naming the themes, and finally, the written process [20]. Initially, all participant interviews were written. Then, when doing thematic analysis, investigators repeatedly read the recorded interview transcripts to familiarize themselves with the findings. The principal investigator independently conducted the coding process for the entire transcript carefully. A total of 25 codes were accumulated into units of the same meaning to form six sub-themes and three main themes. The codes, sub-themes, and themes were reviewed and refined in a repetitive manner through discussion among both researchers. This supported to decrease researcher bias. That was help for different perspective discussion in order to enhance the trustworthiness of study data analysis.

The current study methods have been recorded absolutely with the use of broad details and the including of relevant participants’ quotations to increase the credibility, dependability, transferability, and confirmability of the findings [21].

## Findings

**Fig. 2:**
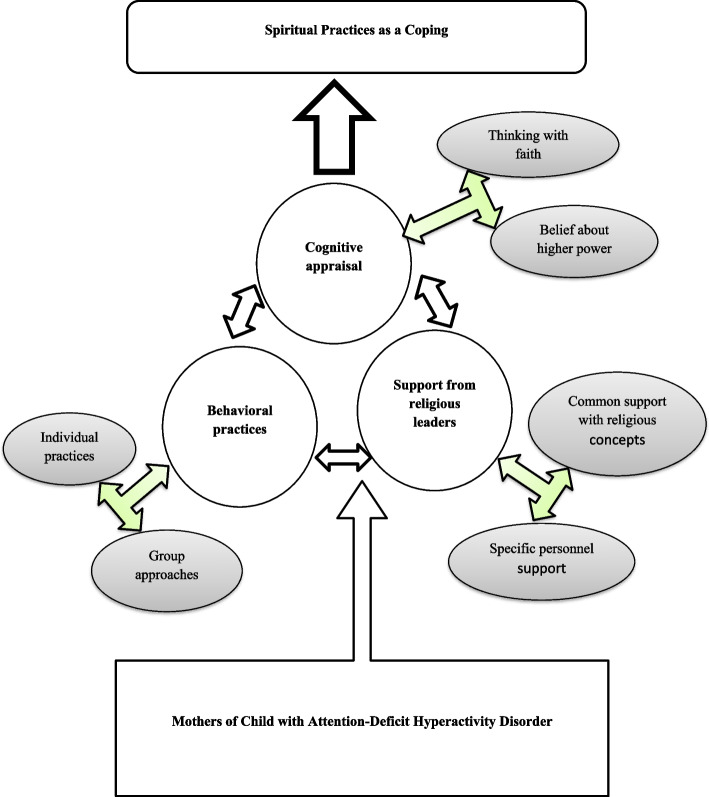
Spiritual practices as coping

All participants of the study were Sri Lankans and had different religious, and social background. Socio demographic characteristics were presented in Table 1. The study findings included an interpretation of the findings and an exploration of the expanding themes. Finally, there were three major themes with six subthemes: 1) cognitive appraisals—thinking about faith and beliefs about higher power; 2) behavioral practices—individual practices and group approaches; and 3) support from religious leaders—common support with religious concepts and specific personnel support. Subthemes were elicited under the three main themes (Table 2). Nevertheless, there were interconnections between all three major themes. Therefore, a final Fig. 2 was generated as follows.

**Table 1:** Demographics characteristics of mothers who having child of with ADHD

	**N**	**%**
Age (mean)	40.18	
**Age**		
30–35	2	9.1%
36–40	9	40.9%
41–45	11	50%
**Religion**		
Buddhist	19	86.4%
Catholic	1	4.5%
Islam	2	9.1%
**Education level**		
Up to Ordinary level	1	4.5%
Passed Ordinary Level	3	13.6%
Passed Advance Level	14	63.6%
Diploma holder	1	4.5%
Graduated	3	13.6%
**Mothers’ occupation**		
Freelance	4	18.1%
Teacher	2	9.1%
Homemaker	9	40.9%
Assistant in Accountce	1	4.5%
Bank teller	1	4.5%
Officer in Agriculture	1	4.5%
Garment worker	1	4.5%
Worker in a shop	1	4.5%
Management Assistant	1	4.5%
Tea worker	1	4.5%
**Age of their child**		
04 years	2	9.1%
05 years	2	9.1%
06 years	3	13.6%
07 years	4	18.1%
08 years	5	22.7%
09 years	2	9,9%
10 years	2	9.9%
11 years	1	4.5%
12 years	1	4.5%
**Sex of their child**		
Male	16	72.7%
Female	6	27.3%

**Table 2:** Framework for coding qualitative findings

Codes	Sub themes	Themes
Faith of the life Karma (things done in the past life) Positive interpreting events Future hopes	Thinking with faith	Cognitive appraisals
Living with values of unseen power Loving to the religion/ God/ power Meritorious thinking with religion Trusting with Gods plan	Beliefs about higher power	
Chanting pirith (sacred words of lord Buddha) Pray for Child Seth kawi (wishes poem) Meditation methods Sacred places Worship	Individual practices	Behavioral practices
Bodhi pooja (treat for scared tree) Worshiping programs Religious discussions Pray sessions	Group approaches	
Gain religious content Prayer pooja Guide for religious rituals	Common support with religious concepts	Support from religious leaders
Social help being a listener Religious counselling Economical support	Specific personnel support

### Theme one—cognitive appraisals

Under this approach, there are two subthemes. Those who are thinking with faith believe in greater power. Mothers’ life events are always binding with their child, and they are given priority for their child. As mothers, they face more psychological distress because of their child’s condition. They always tried to give reasons and explanations for their situations from their religion. The participants explained their situation. It appeared to be a positive coping method for their states.

“I have done good things for others; I always support others. However, it was happened to my child. why do bad events happen to me? It may be faith in my life.” [Interview 03, M03]

Among the 22 mothers, 19 were Buddhists, and they had a thinking pattern regarding the faith of their life. They also believed that these things happened due to their previous karma (good or bad things that they had done in the past), and they also had positive thinking and coping patterns to change their faith. One participant explained,

"I did not expect this kind of illness for my child. However, now it has happened. It has arrived from my son and my previous karma. That is the fate of our life. However, we can change our fate as told by our religion. Therefore, we are always doing meritorious things, and it will help my son to be a healthy child." [Interview 07, M07].

All the participants believed in greater power. They had hope for help from various types of divine powers, such as the power from Lord Buddha, the power from the Bodhi tree, sacred relics, and various gods. Therefore, they always have hopeful eyes for their child's healthy future. One participant expressed:

"We always do Bodhi pooja and follow Buddhist rituals, seeking support from Lord Buddha's power. All gods will always help me and my child. My child will be cured." [Interview 07, M07].

However, these kinds of positive thoughts were helpful for tolerating their conditions and were very effective coping methods for them.

### Theme two—behavioral practices

The impact of behavioural practices incorporated as a major theme among the challenges expressed by participants of the study. Under this theme, there are two subthemes: individual practices and group approaches.

As mothers, they faced emotional challenges. Then, they engage in spiritual behavioral practices as a coping mechanism. All 22 mothers in the study reported using individual spiritual care practices as their coping strategy. One mother explained,

“I engaged every day evening for chanting pirith (Lord buddhas sacred teaching words) and practiced rituals of our tradition, such as seth kawi (wishes poems). I believed it would be helpful to cure my child immediately” [Interview 11, M11].

Another one was explained.

“I always pray for my child with God; I believe that He gives support for my child definitely” [Interview 19, M19].

One mother expressed:

“We went to pilgrims for worship sacred places. We have given alms for monks and nuns. It was help to me tolerate my situation, and it will give wishes for my child” [Interview 05, M05].

Among the 12 out of 22 mothers who practiced meditation as an individual coping method, it was found to be very helpful for them, as expressed in their own words. One participant described her situation as follows:

"I always practice maithree bawanawa (growth of a mind of unconditional love and better benevolence for all sentient beings), which includes an important spiritual practice in Buddhism that ultimately culminates in identification with all beings. It will support me" [Interview 12, M12].

Engaging group practices were reported by 16 out of 22 mothers in the study. They reported their practices with spiritual group practices as their coping method. One mother expressed,

"My sons always like to go to the temple. We always participate in the Saturday bodhi pooja, which is conducted by a monk once a week. However, my son cannot stay seated for a long time, so he walks around the bodhi tree and engages in the activity. I think it is enough for me" [Interview 20, M20].

Another mother explained,

"I try to participate in every Sunday church session to receive blessings from God. My child also sometimes engages in the sessions. It always gives relaxation to the mind" [Interview 19, M19].

#### Theme three—support from religious leaders

There was some major support from religious leaders for mothers to relieve their anxiety. Additionally, they provide social and financial support for mothers. Mothers did not receive support from the government for their social issues. In accordance with the sociocultural situation in Sri Lanka, most individuals seek support from their religious places. The study identified two subthemes under this major theme: common support with religious concepts and specific personnel support. Among the 22 participants, 19 had common support with religious concepts from their religious leaders, regardless of their religion.

*“I had advice from our chief monk at the village temple. He always continued dhamma discussions and speeches related to the faith of life. According to Buddhist concepts, I always got something for me and my family. I always try to follow monthly meditation sessions of the temple. Better advice and paths were learned from venerable monks. I got relaxed from it” [Interview 06, M06].*

As one mother explained,

*“I got more things from church. I leaned from fathers to cope. I always did read scripture and pray with them on behalf of my child. I was involved in singing carols. It gives some help in coping with my situation” [Interview 19, M19].*

Among the 22 mothers, 10 had specific personal support from their religious leaders for coping. It was highly supportive for them, according to their voices. One mother described her practices as follows*:*

*"I received personnel counselling support from Buddhist monks and nuns. They always provided strong words of encouragement to live with hope. Sometimes they dedicated more time to listening to my story, which was very helpful for me. I received more personnel advice and applied those concepts to my child, and it was effective" [Interview 10, M10].*

As another mother mentioned,

*"My husband is a manual worker, and we are facing severe economic issues. We received more financial and social support from our mosque, which provided better support for us. It was very helpful for the continuation of medical follow-up and medications for our child" [Interview 04, M04].*

One of the participants explained as follows:

*"Our temple has a preschool, and the chief incumbent monk is aware of my child's condition. The preschool fees have been waived for my son. The monks and preschool teachers always show kindness towards our child, and our child also enjoys staying in the temple" [Interview 07, M07].*

## Discussion

The findings of the current research illustrate that mothers use spiritual practices as coping mechanisms for mothers of children with attention-deficit hyperactivity disorder. Mothers perceive influences from coping in their minds. They have different cognitive appraisals as coping methods to maintain their mental well-being. These findings are similar to previous studies' findings, such as providing care for children with developmental disabilities with positive and satisfactory measures. This situation may have been generated from the mothers' strengths in terms of psychological aspects and strong spirituality ideas [22, 23]. Some mothers consider having a child with an illness not a burden but rather their faith [22]. Similarly, spirituality is helpful for mothers of children with developmental disabilities [23].

The study participants reported behavioral practices regarding spiritual and religious practices as their coping mechanisms. Several participants practiced praying, engaged in religious practices and rituals, and engaged in meditation. These similar themes have been recorded by other previous researchers looking at the behavioral approaches of religiosity. For example, parents of the child reported that they could reduce their stress and believe that good days will arrive after completing their prayers [24]. In the present study, mothers practiced meditation with mindfulness techniques. A previous study reported similar findings regarding the practice of mindfulness techniques and their impact on reducing the stress of parents and children's behavior problems [25].

The support of religious leaders is a major part of spiritual care. Supporting the client in participating in religiosity and spiritual pursuits includes referring them to clergy, apprising them about available resources, providing material on relevant religious practices, granting them the opportunity to pray and meditate, and supporting them in engaging with religious support and associated activities. These are all parts of providing spiritual care [25]. However, there is no reference system for clergy in the Sri Lankan setting. Nevertheless, the current study includes mothers as caregivers who have practiced that align with other components of receiving significant support from religiosity and spiritual behaviors.

In previous research, support from religious leaders has been reported. Most studies have focused on support from religious leaders for patients. They have also emphasized the importance of spiritual care for the client, which can be provided in two different ways: general spiritual care, which involves being there, understanding, showing compassion, and actively listening. Similar approaches can be offered by anyone at any time [26–28]. The participants in the current study also reported similar practices with religious leaders, such as Buddhist monks and nuns, Catholic fathers, and other religious leaders.

Previous studies have reported on specific spiritual care requirements for clients. If a client has more complex issues, they may require the expertise of well-trained spiritual care counselling professionals, such as chaplains who have received training in Clinical Pastoral Education. These chaplains have a strong background and can provide collegial and informed support in dealing with religious issues that are clinically related to most patients and their families. Chaplains are often consulted and focus primarily on end-of-life care issues, making their services highly valuable [27, 28]. However, in the Sri Lankan context, there are no chaplains or professional spiritual care counselors available to assist patients or their family members. Despite this situation, the study participants received significant support from religious leaders to alleviate their distress.

In conclusion, the study findings and previous literature have explored the spiritual practices as a coping mechanism for mothers who have a child with ADHD. Therefore, this is aligned with the religious meaning-making model [15].

## Limitations of the study

The current study was restricted to a small size of the sample, and it was conducted in one state sector hospital in Sri Lanka.

## Conclusion

Mothers who have a child with ADHD face challenges in coping with their child's illness. They have used different coping methods, such as cognitive appraisal with faith and belief in a higher power, as well as behavioral practices, including individual and group approaches. They also seek support from religious leaders and find comfort in religious concepts and specific personnel support.

The current study discusses the practices of mothers who rely on religious commitment as a coping mechanism while caring for a child with ADHD. The study revealed that these mothers utilize culturally bound religious practices, which they perceive as effective in managing their mental health issues and maintaining a good level of coping.

### Recommendation

The government should focus on establishing child and adolescent clinical settings that are feasible for all areas in Sri Lanka. They should also pay attention to parents' mental well-being and psychosocial support services. The state should be aware of the need to develop and maintain a profession of child and adolescent mental health nurses in Sri Lanka. When considering spiritual care support, they should be aware of the importance of establishing the expertise of well-trained spiritual care counselling professionals, such as chaplains who have received training in Clinical Pastoral Education, in the Sri Lankan health sector. Psychoeducation trainings, such as Cognitive Behavioral Therapy (CBT) and distraction techniques training programs, can be arranged to further help mothers of this kind to cope better with the effects of ADHD.

## Data Availability

All data and materials are available. Please contact the authors for a reasonable request.

## Abbreviations

ADHD: Attention Deficit Hyperactivity Disorder
CBT: Cognitive Behavioral Therapy
